# Qualitatively exploring the impact of a relationship-centered communication skills training program in improving patient perceptions of care

**DOI:** 10.1016/j.pecinn.2022.100069

**Published:** 2022-07-27

**Authors:** Marie C. Haverfield, Robert Victor, Brenda Flores, Jonathan Altamirano, Magali Fassiotto, Merisa Kline, Barbette Weimer-Elder

**Affiliations:** a Department of Communication Studies, San Jose State University, San Jose, CA, USA; b Office of Faculty Development and Diversity, Stanford Medicine, Stanford, CA, USA; c Physician Partnership Program Patient Experience, Stanford Health Care, Stanford, CA, USA

**Keywords:** Communication skills, Qualitative methods, Patient experience, Patient-clinician relationship

## Abstract

**Objective:**

To explore qualitative patient experience comments before and after a relationship-centered communication skills training to understand patient experience, program impact, and opportunities for improvement.

**Methods:**

Qualitative patient experience evaluation data was captured from January 2016 to December 2018 for 483 health care clinicians who participated in the skills training. A random sampling of available open-ended patient comments (*N* = 33,223) were selected pre-training (*n* = 668) and post-training (*n* = 566). Comments were coded for valence (negative/neutral/positive), generality versus specificity, and based on 12 communication behaviors reflective of training objectives.

**Results:**

No significant difference was found in the valence of comments, or generality versus specificity of comments before and after the training. A significant decrease was present in perceived clinician concern. “Confidence in care provider” was the communication skill most frequently identified in comments both pre- and post-training.

**Conclusion:**

Perceptions of interactions largely remained the same following training. Key relationship-centered communication skills require further attention in future training efforts. Measurements of patient satisfaction and engagement may not adequately represent patient experience.

**Innovation:**

This study identified areas for improvement in the training program and offers a model for utilizing patient experience qualitative data in understanding communication training impact.

## 1 Introduction

The quality of communication between clinician and patient plays a critical role in both the actual and perceived care provided. Having the perception of decisional control in one's care, open lines of communication with the clinician, and answers to condition-related questions, produces more positive psychological outcomes in patient populations as compared to those that are not given similar care [[1], [2], [3]]. Other features of communication that predict patient experience with care include clinicians' explanation of symptom causes, perceived clarity, perceived partnership and time spent with patients [[4], [5], [6]]. Taken together, clinician behavior is linked to patient quality of life. As such, much of the success of our health care delivery system depends on a clinician's approach to communicating diagnosis and treatment in a way that involves a patient and reinforces their trust [7].

The relationship-centered care (RCC) framework reflects these efforts by positioning the relationship between patient and clinician as central to patient care [8]. According to the RCC framework this is accomplished through four key principles. Principle 1 emphasizes personhood by encouraging consideration of and appreciation for the values, experiences, and beliefs that the patient and clinician each bring to the clinical encounter. Principle 2 encourages emotional presence from clinicians. Rather than removing emotions from the health care experience, emotional presence promotes empathetic interactions that aid in patient expressions of emotion, facilitate better navigation of patient health, and improve patient perceptions of care [[9], [10], [11]]. Principle 3 acknowledges the reciprocal influence between patient and clinician. According to this principle, patient-clinician interactions impact the experiences of both parties involved. Finally, Principle 4 emphasizes the importance of approaching patient interactions with authenticity rather than exchanging superficial information based on an assigned task or role. According to the RCC framework, the genuine effort to develop, foster, and maintain the relationship reflects a moral foundation. Each of these principles are enacted through strong interpersonal communication skills or relationship-centered communication [12]. Notably, the philosophy of RCC extends beyond the clinician-patient relationship to include clinician-clinician relationships, and the clinician's relationship with the broader community [8].

The RCC philosophy has been adopted by multiple institutions, including the Academy of Communication in Healthcare (ACH) [12]. Informed by the RCC philosophy, the ACH developed a specific relationship-centered communication training program to educate health care professionals as well as offer opportunities for health care systems to adapt and customize the relationship-centered communication training to reflect the needs of their own respective organizations [13]. The ACH evidence-based curriculum focuses on helping clinicians to build immediate rapport (RCC Principle 1); use emotion and compassion to engage in authentic relationship-centered encounters (RCC Principle 2 and 4); confirm mutual understanding (RCC Principle 3); establish two-way information sharing (RCC Principle 3); and improve patient health, experience, and overall quality of care (RCC Principle 4).

In partnership with the ACH, a northern California Academic Medical Center adapted the relationship-centered communication training into a program titled Advancing Communication Excellence at Stanford, or ACES. The ACES training program is a facet of a larger organizational model to foster a relationship-centered institutional culture known as the ALPS model. The ALPS model (**A**sk, **L**earn, **P**artner, **S**tudy, synthesize, and support) [14]^,^ features various forms of educational programming including consultations, workshops, and coaching sessions for affiliated faculty. The model reflects an effort to continually evaluate programming to ensure clinician communication skill-building needs are met and enhanced, informed by partnerships with interdisciplinary experts, clinicians, and patients. In line with this transformational vision, all ACES training efforts are evaluated from multiple viewpoints to identify opportunities for improving educational programming.

This study explored ACES, aimed at enhancing clinician relationship-centered communication skills, through an analysis of survey-based patient experience comments. Numerous studies have examined patient experience survey data to evaluate clinician performance, often resulting in a ‘top box’ phenomenon, where most patient reports skew positively, making it more challenging to discern actual experience and behavior [15,16]. Few studies however, have considered the qualitative comments provided by patients in the evaluation of clinician performance and care management [17,18]. Traditionally, qualitative comments afford the ability to capture an authentic, descriptive perspective that self-reported quantitative scales typically do not provide. Therefore, qualitative comments may provide greater context for the care received. Based on the objectives of the ACES training, and in line with the reflexivity of the ALPS model, we set out to analyze patient experience qualitative survey data to understand the patient care experience, training program impact, and identify opportunities for improving future relationship-centered communication programming based on patient insights. Collectively, we anticipated patient comments post-training to be more positive, with greater specificity to the care received, that highlight clinician communication skills representative of the training objectives, which aim to promote RCC.

## 2 Methods

### 2.1 Training/intervention

**Fig. 1:**
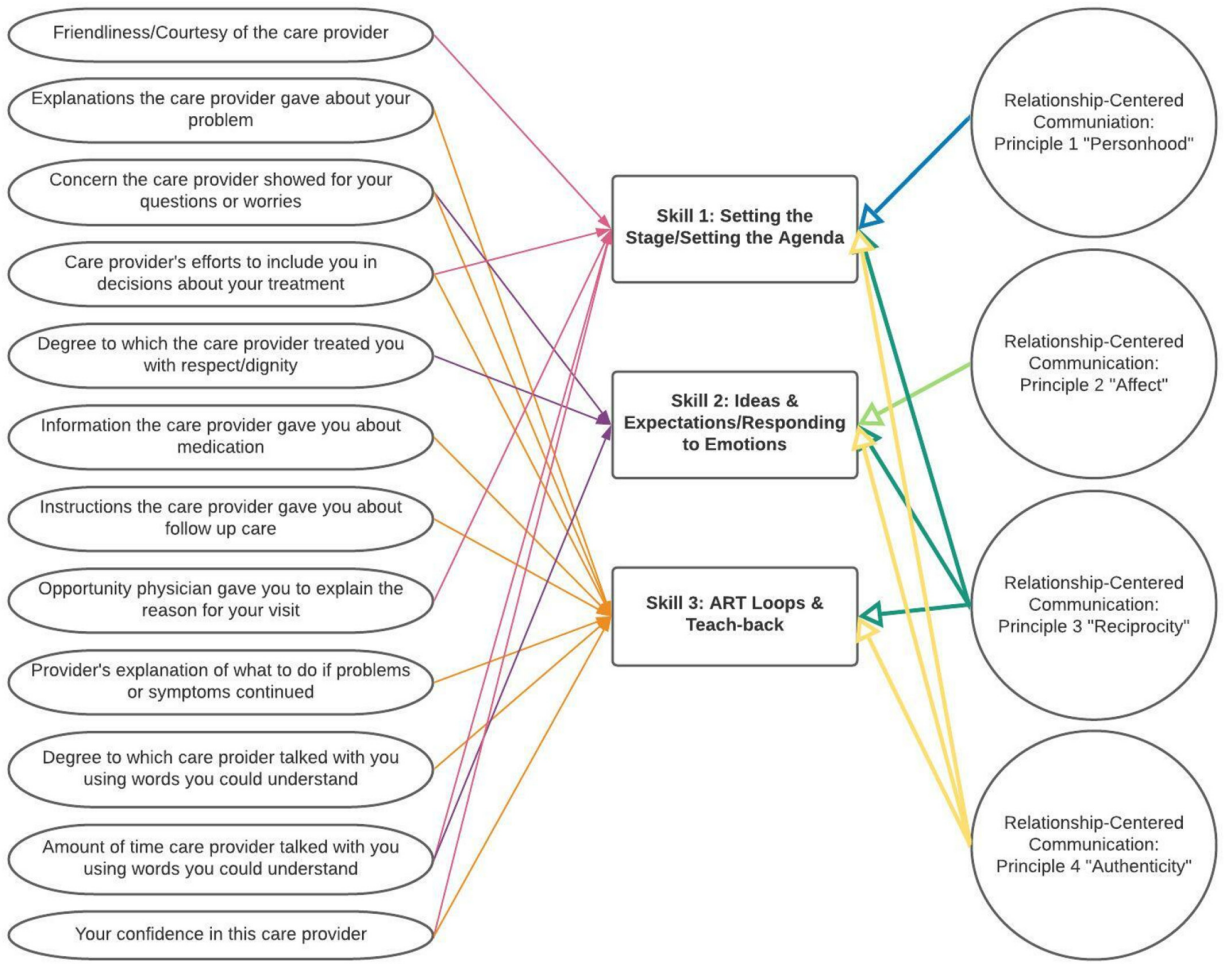
Communication Behaviors, ACES Macro Skills, and RCC Mapping.

The relationship-centered communication skills training, ACES, consists of an 8-h in-person workshop that serves as a foundational skill-building training for clinicians. Open to all health care system clinicians, ACES workshops are free of charge, are held multiple times per month to accommodate clinician availability, and typically train 12 clinicians in a single workshope. The course covers three primary content areas (macroskills) that contain several microskills. Adapted from the relationship-centered communication evidence-based curriculum [13]^,^ each of these macroskills map onto the four principles of the RCC framework (see Fig. 1). The primary content areas include: 1. Setting the Stage/Setting the Agenda (RCC Principle 1, 3, & 4); 2. Ideas and Expectations/Responding to Emotions (RCC Principle 2, 3, & 4); 3. Ask-Respond-Tell (ART) Loops and Teach-back (RCC Principle 3 & 4). Throughout the duration of the ACES training, points of evaluation data were gathered from attendees. Patient experience evaluation surveys pre- and post-training were also reviewed, to determine program effectiveness and areas for improvement [19]. Clinicians from this study sample participated in a single ACES workshop and did not take part in any other ALPS related programming.

### 2.2 Patient experience survey

The patient experience survey is routinely administered via regular mail and e-survey. Among patients who receive care, 25 are surveyed by regular mail per clinician per month. All other patients who receive care are sent an e-survey. The e-survey is administered through an integrative digital tool that allows patients to securely access elements of their health information. Patients receive the e-survey the day after their visit or if sent by regular mail, approximately one week after the visit. Patients under 18 years of age are not surveyed.

Patient experience survey comments were collated pre- and post-training between January 2016 and December 2018 to gain insights on the effect that relationship-centered communication training had on patient experience and perceived quality of care. Specifically, patient experience evaluations for ACES participating clinicians were obtained from 6-months pre- and post their respective ACES training. Approximately 33,223 patient experience evaluations were completed during that period for participating clinicians, with an average of 53.5 patient evaluations per clinician and a median of 43 comments.

On the survey, patients are asked both general questions related to the clinic visit and more specific questions about the attending clinician. For the purposes of this study, patient responses to the prompt, “Describe good or bad experience,” were isolated from patient experience surveys. On the patient evaluation survey this prompt is presented to patients following a series of Likert scale questions pertaining to the attending clinician, with the clinician's name appearing at the top of the survey. The series of self-report Likert scale questions presented to patients prior to the identified prompt refer to the experience the patient had with their care (i.e., physician explained patient medical condition, physician confirmed patient understanding). From the 33,223 evaluations, we randomly selected five pre- and five post-workshop comments for each clinician. Sample selection was due to the range in available patient experience surveys per clinician. On average, clinicians had five patient experience surveys available both pre- and post-workshop participation. Further, in an effort to explore the utility of patient experience survey open-ended data, no inclusion or exclusion criteria was applied to the random selection of responses. Taken together, this approach resulted in a total of 668 pre-workshop comments and 566 post-workshop comments. Comments averaged approximately 24.25 words and ranged from 1 to 305 words.

### 2.3 Coding scheme

Selected comments were de-identified and entered into an excel spreadsheet for coding purposes. Each row in the spreadsheet represented a single patient comment and a column was created to enter a single code for valence and the generality/specificity of the comment. In line with the goal of the ACES workshop and broader ALPS model, we included two additional columns for capturing primary and secondary codes reflective of specific communication behaviors. Codes were created based on the survey Likert-scale questions. Two coders (BF, RV) were trained to analyze patient comments. Coders each received the coding spreadsheet of deidentified patient comments, equipped with the descriptions for coding valence, specificity or generality of comment, and list of communication behavior categories.

For valence, coders were instructed to determine whether the comment demonstrated a negative experience (e.g., “*I just didn't get a good sense of empathy or even any emotion from her.*”), neutral experience/miscellaneous information (e.g., “*The care provider has since responded to personal e-mail with direction and comments.*”), or positive experience (e.g., “*Very excellent provider. Would not change it for anything. Yes I would refer to others.*”), coded as −1, 0, and 1 respectively.

Adapted from the work of Iversen et al. (2014), we also coded for the specificity or generality of patient comments. Coders rated whether the comment identified a specific aspect of care (e.g., “*Did not know how surgery was unsuccessful. Blamed it on referring doctor. Very unprofessional in determining why I was still having pain 1 year after surgery.*”), or was more general in nature (e.g., “*Good experience*”), coded as 1 and 2 respectively [20].

Finally, comments were coded based on 12 communication behavior categories adapted from the aforementioned self-report Likert scale questions. The 12 communication behavior categories are indicative of relationship-centered communication and ACES skill-building content (see Fig. 1). Each behavior was assigned a numerical identifier for coding purposes: 1) Friendliness/courtesy of care provider, 2) Explanations provider gave about problems, 3) Concern provider showed for questions/worries, 4) Provider efforts to include patient in decision making, 5) Provider treated patient with respect/dignity, 6) Information about medication, 7) Instructions about follow up care, 8) Opportunity to explain reason for visit to provider, 9) Explanation of what to do if problems symptoms continued, 10) Provider used words that were easy to understand, 11) Amount of time spent with provider, and 12) Confidence in care provider. The behavior category, “Confidence in care provider”, encompasses a range of behaviors that convey to the patient that the clinician is capable in managing patient care [21]. Because a single comment could include multiple behaviors, coders were instructed to independently assign each comment a primary category (or behavior) and then, if applicable, code for any secondary behaviors also present in the patient comment. The primary code reflects the communication behavior most represented by the comment. For example, a comment that described clinician attentiveness to patient's feelings would be coded as ‘3’ reflecting the behavior category “Concern provider showed for questions/worries”. Secondary behaviors could include additional behaviors such as tone of voice, efforts to gather all patient concerns, and the ability to share bad news [22,23]. The presence of a behavior did not specifically refer to a positive demonstration of that behavior. For example, a comment could refer to the lack of confidence in a clinician and be categorized under the behavior ‘Confidence in care provider’. A miscellaneous category was added to capture comments that did not directly refer to any of the communication categories. Codification of comments confirmed the communication behavior present and afforded the ability to establish contextual details about the patient experience to aid in program evaluation and identifying opportunities for improvement.

### 2.4 Coding procedures

After reviewing the spreadsheet and coding criteria, coders independently coded the first 20 pre- and post-workshop patient satisfaction survey comments, totaling 40 comments. Following the initial round of coding, coders met with the first author (MH) to discuss codes, confirm reliabilities, and resolve any discrepancies. The same process was followed for two more rounds of coding 20 comments for a total of 120 comments coded, or approximately 20% of the codes. Following the third round of coding, coders received instruction to complete coding for remaining comments. Coder reliability for valence and the generality or specificity of the comment was assessed using intraclass correlation coefficient [24]. To establish a mean score of valence and generality versus specificity, coders met to discuss disagreement and all discrepancies were resolved to achieve 100% reliability. Discrepancies across behavior coding were also discussed, during each round of coding, to ensure 100% reliability in assigned communication behavior categories.

### 2.5 Data analysis

Five analytical approaches were used to explore the impact of the ACES training program on clinician communication per patient experience survey responses. A paired samples *t*-test was used to explore pre-post differences in response valence. A McNemar test, and the less conservative Cochran's Q, were used to determine differences in pre-post patient responses coded for generality versus specificity. A chi-square test examined differences in reported communication behaviors between pre- and post-training, and descriptive reports of behavior frequency conveyed the presence or absence of behaviors identified by patients. All data was analyzed using SPSS v28.

## 3 Results

Participating clinicians (*N* = 483) identified as female (62.9%), and White (31.5%), with 11–20 years of clinical experience (34%) working more than 80% of clinical time, at a Clinical Assistant Professor rank (23.6%) in medicine (26.3%). Most clinicians had no prior communication course experience (76.8%).

Patients included in the survey comment data (*n* = 1254) predominantly identified as female (59.9%), Caucasian (46.9%), followed by Asian (34.9%), Hispanic/Latino (4.3%), Two or more [non-Underrepresented Minorities] (1.1%), Black/African American (0.9%), Native Hawaiian/Pacific Islander (0.7%), American Indian/Alaska Native (0.2%), and 10.9% declined to state. The average age of patients was 58.9 (*SD* = 17.45), ranging from 18 to 87 years of age. Patient visits occurred within a range of medical divisions including but not limited to neurology, dermatology, anesthesia, and sports medicine.

### 3.1 Valence

Coding analysis for both pre-workshop valence (ICC = 0.79) and post-workshop valence (ICC = 0.74) demonstrated acceptable reliability, supporting coding scheme validity. After clinicians underwent the ACES training program there were no significant differences in comment valence (*t*(1.15) = 533, *p* = .13) between pre-workshop and post-workshop comments. Consistent with previous self-report evaluation research, the majority of comments both pre- (*n* = 581, 85%) and post-training (*n* = 464, 82%) skewed positively. Although not significant, the proportion of negative comments declined from 10.2% pre to 8.8% post, and there was an increase in neutral/miscellaneous comments from pre (2.8%) to post (9.2%). Taken together, assumptions that post-training responses would be more positively valanced was not supported.

### 3.2 Generality versus specificity

Pre-workshop comment type (general vs specific; ICC = 0.92) and post-workshop comment type (ICC = 0.84) demonstrated high reliability among coders. In terms of the general versus specific nature of a patient comment, an exact McNemar's test determined that there was no statistical difference in the proportion of general versus specific responses pre- and post-workshop, *p* = .12. McNemar's test was followed by a less conservative analysis to determine difference in proportion, Cochran's Q, which also found no statistical difference between pre-post general versus specific comments X^2^(2) = 2.85, *p* = .091. Thus, our prediction that more specific comments would be evident post-training, was not supported.

### 3.3 Communication behavior

We then analyzed patient experience comments for the 12 communication behaviors to determine change in comments pre- and post-training. Chi-square tests found no significant difference in eleven of the 12 communication behaviors (see Table 1). The significant change found was a decrease in the ‘Concern provider showed for questions/worries’ between pre- and post-training comments, X^2^ (1, *N* = 1234) = 5.08, *p* = .024.

**Table 1:** Communication Behavior Categories Frequency.

Category		Pre-Intervention		Post-Intervention		Sig.
		n	%	n	%	*p* < .05
1	Friendliness/courtesy of care provider	198	29.6%	157	27.7%	0.462
2	Explanations provider gave about problems	59	8.8%	42	7.4%	0.367
3	Concern provider showed for questions/worries	56	8.4%	29	5.1%	0.024
4	Provider efforts to include patient in decision making	7	1.0%	8	1.4%	0.559
5	Provider treated patient with respect/dignity	16	2.4%	16	2.8%	0.635
6	Information about medication	2	0.3%	4	0.7%	0.305
7	Instructions about follow up care	16	2.4%	11	1.9%	0.589
8	Opportunity to explain reason for visit to provider	1	0.1%	0	0.0%	0.357
9	Explanation of what to do if problems symptoms continued	0	0.0%	0	0.0%	0
10	Provider used words that were easy to understand	10	1.5%	8	1.4%	0.903
11	Amount of time spent with provider	48	7.2%	40	7.1%	0.936
12	Confidence in care provider	249	37.3%	236	41.7%	0.113
13	Miscellaneous	6	0.9%	15	2.7%	0.029

In addition to determining significant change over time, we also report the frequency of patient experience communication behaviors coded across comments (Table 1), as a proxy for the primary relationship-centered communication skills content from the ACES workshop (Fig. 1). Although not significant, we report the frequencies to provide descriptive detail to our understanding of the presence (or absence) of the 12 communication behaviors according to patient accounts of their clinic visit experience.

After clinicians underwent the ACES training program the frequency of applicable communication behaviors largely, but not significantly, decreased according to patient comments. Three communication behaviors demonstrated an increase or consistent frequency pre- to post-training: “Provider efforts to include patient in decision making”, “Provider treated patient with respect/dignity”, and “Information about medication”. Examples of these comments include, “*He listened to my problem, examined me, and described various options. I made a choice and he ordered a prescription at my pharmacy*” and “*She truly cares about her patients! She makes me feel like an individual not just a number*”. “Confidence in care provider” was the primary skill most often mentioned in pre- and post-workshop comments (37.3% and 41.7% respectively). Notably, 2 of the 12 communication behavior categories did not appear in any comments following the ACES training program: “Opportunity to explain reason for visit to provider” and “Explanation of what to do if problem symptoms continued”.

Comments that informed “Confidence in care provider” had little to do with long-term foci of care, such as the medicine a patient would be receiving or information on follow-up treatments, skills that were much less evident in comments. Consistent with previous research, comments focused more on the way care was handled in the moment; the friendliness, respect, and thoroughness that a clinician demonstrated in their communication with the patient [25]. Another notable finding is the extent to which patients noted the perceived time spent with the clinician. Patient perceptions of receiving ample time to communicate with the clinician can contribute to improvements in patient satisfaction and fewer unmet concerns [26,27]. Together, these findings align with the principles of the RCC framework.

As mentioned, additional secondary codes were assigned to comments that encompassed multiple communication behaviors. With the majority of pre- and post-comments reflecting the communication behavior “Confidence in care provider”, we examined the secondary codes most often found in conjunction with the “confidence” primary code, or a skill pairing. The skill pairing aimed to determine what attributes are most associated with “Confidence in care provider”.

**Fig. 2:**
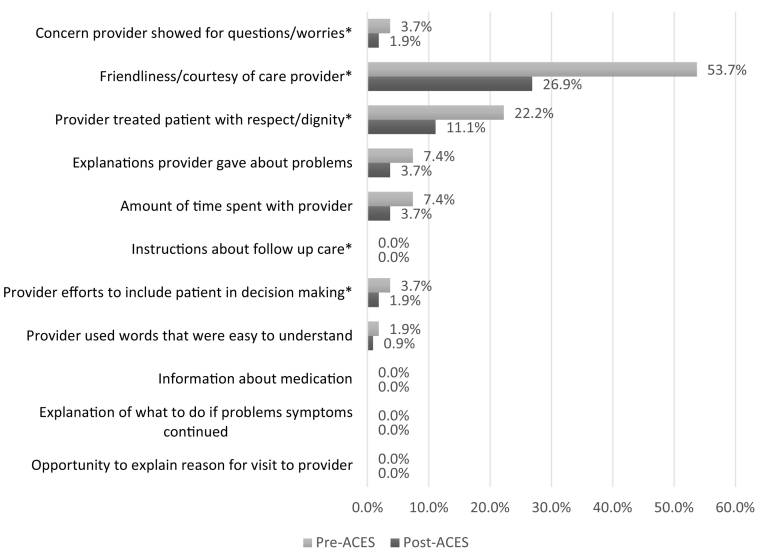
Pre- and Post-ACES Secondary Skill Pairing with “Confidence in care provider”.

Prior to the ACES training four secondary communication behaviors were most frequently paired with “Confidence in care provider”: “Friendliness/courtesy of care provider”; “Provider treated patient with respect/dignity”; “Explanations provider gave about problems”; and “Amount of time spent with provider” (see Fig. 2). Examples of patient responses that reflect this skill pairing include, “*Dr. actually TALKED to me for an hour…he also caught something on my ct scan and called me himself to tell me about it*” and “*Seemed ‘prissy’ and judgmental*”.

Following ACES training, three secondary codes were most frequently paired with “Confidence in care provider”: “Provider treated patient with respect/dignity”; “Concern provider showed for questions/worries”; and “Amount of time spent with provider”. Related examples for these skill pairing comments include, “*Dr. was professional, compassionate & concerned. He answered ALL my questions*” and “*Dr. is such an awesome doctor and also a very compassionate person. I am touched by her patient relationship skills*”. Across all behaviors, “Friendliness/courtesy of care provider” and “Provider treated patient with respect/dignity” appeared most frequently as a secondary code.

## 4 Discussion and conclusion

### 4.1 Discussion

This study explored the impact of ACES and potential opportunities to improve the training program based on patient care experience comments gathered pre- and post-ACES. We anticipated comments post-training to be more positive when compared to pre-ACES responses however, there was no significant change between pre- and post-comment valence. The non-significant finding that positively valanced comments decreased from pre- to post-training is surprising given the goal of the ACES training program: to optimize the patient experience by enhancing clinician relationship-centered communication skills. While the frequency of negative comments slightly decreased from pre- to post-training (2.6%), a more notable change, albeit not significant, was the increase in neutral/miscellaneous comments (4.7%). We speculate that the reason for this change may be due to patient expectations being met, resulting in no positive *or* negative stance regarding the care provided. Another possibility is that clinicians and patients are collaborating more when discussing patient health care management post-ACES training. As such, the patient might perceive the evaluation as both a reflection of themself as well as of the clinician and approach responses to the care experience prompt more neutrally. It is also important to acknowledge that clinicians participating in ACES are largely those already invested in improving their relationship-centered communication skills, which likely explains the high frequency of positive comments found in our data sample.

We also anticipated post-ACES comments to demonstrate greater specificity regarding the care received when compared to pre-ACES responses. Again, we found no significant difference in the proportion of general versus specific pre- and post-comments. We note that the nature of patient experience survey responses identified as specific are often reflective of two extremes, highly favorable or unfavorable. Not all specific comments are negative (63% positive and 52.8% positive respectively) however, the majority of negative comments are specific (pre = 93.8%, post = 100%). The slight but not significant increase in more general comments regarding the care experience could be attributed to patient visit expectations being met whereas, the decrease in specific comments may represent a possible improvement in patient perceptions of care.

Taken together, findings related to valence and the generality or specificity of comments suggest that more research is needed to determine the extent to which ACES significantly enhances patient care. Adaptations to the ACES training program, such as adding a booster session, could facilitate more significant improvements in both actual and patient-perceived care experience over time. In addition, the evaluation prompt used to gather feedback and determine patient satisfaction and engagement may not be sufficient in effectively eliciting candid responses. Prompts that ask patients to describe *how* the clinician communicated information—particularly in relation to RCC principles, or why they will or will not continue to engage with a particular clinician may be more fruitful for capturing specific details of the care experience that are meaningful to the patient and for confirming the presence of relationship-centered communication behaviors.

Based on the coding results of the 12 communication behaviors, it was evident that numerous aspects of communication are important to building the clinician-patient relationship. Only one communication behavior, “Concern provider showed for questions/worries” was significantly different pre- and post-training. Specifically, patient comments post-training had fewer mentions of clinician concern for patient questions and worries, yet it was among the most frequently paired skills with “Confidence in care provider”. A possible reason for the decrease could be attributed to more clinicians, post-training, engaging in behaviors such as agenda setting, where they purposefully gather patient needs and goals at the start of the visit. By acknowledging and setting expectations about that information upfront, patients may feel that there are less concerns displayed by the clinician but rather more actionable steps to address items on the visit agenda, which may also result in a reduction in patient perceptions of unmet concerns [26].

We also reported the frequencies of behaviors that were not significant, to add descriptive detail to our understanding of the presence (or absence) of the 12 communication behaviors identified by patients. The most frequently coded communication behavior, “Confidence in care provider”, was gauged by overt enthusiasm for the medical skill of a mentioned clinician, a stated desire to continue care under the clinicians' supervision, and intentions to adhere to a prescribed treatment plan. The most frequent secondary behaviors identified in both pre- and post-ACES comments (“Friendliness/courtesy of care provider” and “Provider treated patient with respect/dignity”) suggest that these features of communication are the most central to the quality of the connection made between patient and clinician. These communication behaviors also align with each of the three macroskills of the ACES programming. From this, it appears that the current ACES training program reinforces relationship-centered communication skills that are already largely present among clinician behaviors. Future ACES content, and content for similar communication-based trainings, should continue to emphasize that clinicians demonstrate respect and overall friendliness in order to enhance the patient's care experience.

Findings also point to opportunities for improving ACES. Patient comments help to highlight which relationship-centered communication behaviors could benefit from additional focus, offer evidence for the importance of adopting these behaviors, and provide examples of behaviors that were perceived as inhibiting the clinician-patient relationship. Although some skills may be applicable to more unique patient circumstances (i.e., “Explanation of what to do if problem symptoms continued’), it is still important to ensure that clinicians are equipped to effectively navigate these interactions with a patient. Given the decrease in frequency or absence of communication behaviors, evidence suggests that ACES programming may benefit from supplementing initial training with booster sessions and/or online content to continue skill-building. None of the clinicians in the study sample engaged in additional ALPS educational modalities such as consultations and coaching. Coupling modalities with the workshop could promote more positive change. These specific communication behavior findings can also be incorporated into the transformative process reflected in the ALPS model, to generate change and opportunities that foster an organizational relationship-centered culture. Additional research is necessary to determine how well skills are enacted in practice.

The relationship-centered communication skills that appeared most prominently in patient comments (i.e., confidence in provider, friendliness of provider, explanations provider gave) are qualities that are not exclusive to certain specialties, individuals, or healthcare organizations. Consistent with the RCC philosophy, relationship building skills are beneficial for interprofessional relationships, clinician-community relationships, and a clinician's relationship with self-awareness [8]. Further, relationship-centered communication skill building programs are not exclusive to individuals with a medical doctorate. Thus, training programs like ACES can be impactful and broadly applicable to comprehensive healthcare teams. Programmatic research to examine the effectiveness of a similarly trained cohort of other hospital staff constituents may be an important next step in fostering a relationship-centered culture.

Some limitations exist when analyzing qualitative patient comments. Our findings are limited by the small, and specific, clinician sample. With only 483 clinicians rated, all of whom are affiliated with the same health care institution, the applicability of our findings to larger, or more diverse, clinician populations may be limited. Furthermore, due to the timing of when training was received and evaluations completed, we are unable to look directly across ACES training on a patient-specific level. In addition, it is likely that patients who took the time to complete evaluation forms are polarized and either wish to express their extreme satisfaction or dissatisfaction with the care received. To this point, the prompt for the data set encourages patients to identify good and bad care, which may result in other care experiences not being adequately captured. Our approach to analyzing the qualitative data presents more of a deductive approach. Although the data is rich, a less efficient and more inductive approach, such as semi-structured interviews, could make for richer data. The usage of multimodal data-collection formats, alongside patient satisfaction surveys, may elicit data that more precisely informs points of need.

### 4.2 Innovation

Collectively, findings support the utility of patient perspectives in research and advance opportunities for future programming. For clinicians, this work points to specific communication behaviors that patients frequently perceive as highly valuable and supports the importance and need for adopting and enhancing relationship-centered communication skills. Communication is bi-directional. Therefore, exploring how the ACES training program increases patient involvement is an important direction for future research.

For the medical education and academic community, developing and implementing communication skills training programs, this study outlines a process of using qualitative patient experience data to better understand training impact and opportunities for improvement. The methodological approach of this research enables identification of more descriptive patient preferences regarding clinician communication when compared to self-report questionnaires. Further, analysis of open-ended patient evaluation responses may be a more efficient way of gathering descriptive data when compared to conducting interviews and/or focus groups. Perhaps most importantly, this research facilitates consideration and incorporation of patient feedback towards adaptations of training efforts to ensure more immediate and positive change to practice. Relatedly, evaluation tools may benefit from more meaningful patient open-ended prompts, specific to RCC principles, to better understand the care experience and to ultimately learn from patients to improve quality of care.

Finally, at an organizational level, study findings present an opportunity to identify other outlets for promoting RCC within a system. For example, a colleague-to-colleague relationship-centered communication workshop may further aid in facilitating an organizational culture shift. Curriculum that focuses on one specific skill, such as listening, may also be fruitful for organizations in order to enhance individual awareness, self-management, social awareness, and broader relationship building. Moreover, relationship-centered communication programming geared towards organizational leaders and upper management may enhance mentorship and system-level processes that can ultimately enable policy-level change.

### 4.3 Conclusion

Training programs for clinicians that are focused on building relationship-centered communication skills, like the ACES program, appear to reinforce positive qualities in the clinician cohort that are likely to contribute to numerous benefits in healthcare delivery. Our findings suggest minimal change in patient experience following the ACES training and link patient-perceived confidence in their clinician to the overall strength of the clinician-patient relationship. Confidence in care is built upon the quality of communication delivered by clinicians and often includes a combination of communication behavior strategies. Findings also draw attention to the tools used for evaluating clinician performance and care management and demonstrate the potential utility of patient qualitative feedback on medical education curriculum.

## Disclosures

All authors are directly or indirectly employed by the site in which data was collected and analyzed.

## Confirmations

All patient/personal identifiers have been removed or disguised so the patient/person(s) described are not identifiable and cannot be identified through the details of the story.

## Funding

This research did not receive any specific grant from funding agencies in the public, commercial, or not-for-profit sectors.

## Declaration of Competing Interest

The authors declare the following financial interests/personal relationships which may be considered as potential competing interests:

Marie Haverfield reports a relationship with Stanford Health Care that includes: consulting or advisory. Robert Victor reports a relationship with Stanford Health Care that includes: employment. Brenda Flores reports a relationship with Stanford Health Care that includes: employment. Jonathan Altamirano reports a relationship with Stanford Health Care that includes: employment. Magali Fassiotto reports a relationship with Stanford Health Care that includes: employment. Merisa Kline reports a relationship with Stanford Health Care that includes: employment. Barbette Weimer-Elder reports a relationship with Stanford Health Care that includes: employment.

## References

[1] Arora N.K. (2003). Interacting with cancer patients: the significance of physicians’ communication behavior. Soc Sci Med.

[2] Schattner A., Bronstein A., Jellin N. (2006). Information and shared decision-making are top patients’ priorities. BioMed Central.

[3] Shay L.A., Lafata J.E. (2015). Where is the evidence? A systematic review of shared decision making and patient outcomes. Med Decis Mak.

[4] Jackson J.L., Chamberlin J., Kroenke K. (2001). Predictors of patient satisfaction. Soc Sci Med.

[5] Sholz V., Lange S., Rosenberg B., Kromrey M.L., Syperek A., Hosten N. (2019). Identifying communication-related predictors of patient satisfaction in a briefing prior to contrast-enhanced computed tomography. Ins Imag.

[6] Stone M. (2003). What patients want from their doctors. BMJ..

[7] Gulbrandsen P., Clayman M.L., Beach M.C., Han P.K., Boss E.F., Ofstad E.H. (2016). Shared decision-making as an existential journey: aiming for restored autonomous capacity. Patient Educ Couns.

[8] Beach M.C., Inui T. (2006). Relationship-centered care. J Gen Intern Med.

[9] Beckman H.B., Frankel R.M. (2003). Training practitioners to communicate effectively in cancer care: it is the relationship that counts. Patient Educ Couns.

[10] Halpern J. (2001).

[11] Eide H., Frankel R., Haaversen A.C., Vaupel K.A., Graugaard P.K., Finset A. (2004). Listening for feelings: identifying and coding empathic and potential empathic opportunities in medical dialogues. Patient Educ Couns.

[12] Barden A., Giammarinaro N., Fornari A., Cerise J.E. (2019). Impact of a faculty development course on relationship-centered communication skills. J Health Commun.

[13] Chou C.L., Cooley L. (2017).

[14] Weimer-Elder B., Kline M., Schwartz R. (2022). Building a relationship centered culture in healthcare: an organizational framework for transformation. Physician Leadersh J.

[15] Indovina K.A., Keniston A., Manchala V. (2021). Predictors of a top-box patient experience: a retrospective observational study of HCAHPS data at a safety net institution. J Patient Exp.

[16] Lee M.O., Altamirano J., Garcia L.S., Gisondi M.A., Wang N.E., Lippert S. (2020). Patient age, race and emergency department treatment area associated with “Topbox” Press Ganey scores. West J Emerg Med.

[17] Santuzzi N.R., Brodnik M.S., Rinehart-Thompson L., Klatt M. (2009). Patient satisfaction: how do qualitative comments relate to quantitative scores on a satisfaction survey?. Qual Manag Health Care.

[18] Kilaru A.S., Meisel Z.F., Paciotti B., Ha Y.P., Smith R.J., Ranard B.L. (2016). What do patients say about emergency departments in online reviews? A qualitative study. BMJ Qual Saf.

[19] Altamirano J., Kline M., Schwartz R., Fassiotto M., Maldonado Y., Weimer-Elder B. (2021). The effect of a relationship-centered communication program on patient experience and provider wellness. Patient Educ Couns.

[20] Iversen H.H., Bjertnæs Ø.A., Skudal K.E. (2014). Patient evaluation of hospital outcomes: an analysis of open-ended comments from extreme clusters in a national survey. BMJ Open.

[21] Ranjan P., Kumari A., Chakrawarty A. (2015). How can doctors improve their communication skills?. J Clin Diagn.

[22] Barrier P.A., Li J.T., Jensen N.M. (2003). Two words to improve physician-patient communication: What else?. Mayo Clin.

[23] Adebayo P.B., Abayomi O., Johnson P.O., Oloyede T., Oyelekan A.A. (2013). Breaking bad news in clinical settings—health professionals’ experience and perceived competence in Southwestern Nigeria: a cross sectional study. Ann Afr Med.

[24] Courtright J.A. (2014).

[25] Shah A.M., Yan X., Tariq S., Ali M. (2021). What patients like or dislike in physicians: Analyzing drivers of patient satisfaction and dissatisfaction using a digital topic modeling approach. Inf Process Manag.

[26] Brock D.M., Mauksch L.B., Witteborn S., Hummel J., Nagasawa P., Robins L.S. (2011). Effectiveness of intensive physician training in upfront agenda setting. J Gen Intern Med.

[27] Johnson R.L., Sadosty A.T., Weaver A.L., Goyal D.G. (2008). To sit or not to sit?. Ann Emerg Med.

